# The international staging system improves the IPI risk stratification in patients with diffuse large B-cell lymphoma treated with R-CHOP

**DOI:** 10.1038/s41598-017-13254-x

**Published:** 2017-10-19

**Authors:** Xiaolei Wei, Xiaoxiao Hao, Lizhi Zhou, Qi Wei, Yuankun Zhang, Weimin Huang, Jialin Song, Ru Feng, Yongqiang Wei

**Affiliations:** 1 Department of Hematology, Nanfang Hospital, Southern Medical University, Guangzhou, China; 2 Department of Biostatistics, School of Public Health, Southern Medical University, Guangzhou, China

**Keywords:** Prognostic markers, B-cell lymphoma

## Abstract

The international staging system (ISS), based on serum beta-2 microglobulin and albumin, is used to predict survival in multiple myeloma, but its prognostic significance in diffuse large B-cell lymphoma (DLBCL) remains unknown. Herein, we retrospectively analyzed 215 de novo DLBCL patients. According to ISS, there were 90 of 215 (41.9%) patients in stage I, 98 of 215 (45.6%) in stage II and 27 of 215 (12.6%) in stage III group. Patients with ISS stage II/III showed shorter overall survival (OS) and event free survival (EFS) than those with stage I treated with R-CHOP (*p* = 0.012 and *p* = 0.043, respectively), but not those treated with CHOP regimen (p > 0.05). Multivariable analysis revealed that ISS, independent of IPI, indicated different survival in both OS (HR, 5.690; 95% CI, 1.270–25.495, p = 0.023) and EFS (HR, 2.116; 95% CI, 1.005–4.455, p = 0.049) in DLBCL patients treated with R-CHOP. ISS could identify patients with better outcome in intermediate-high/high IPI risk patients (*p* < 0.05). Our data suggests that advanced ISS stage is associated with inferior outcome in DLBCL patients treated with R-CHOP. ISS could identify a subgroup of DLBCL patients with superior outcome from high IPI risk patients, which may help to avoid intensive therapy.

## Introduction

Diffuse large B-cell lymphoma (DLBCL) is the most common subtype of non-Hodgkin’s lymphoma (NHL) with different clinical behaviors and response to treatment^1^. Although the addition of rituximab into CHOP regimen (cyclophosphamide, doxorubicinm vincristine and prednisone) has dramatically improved the survival of DLBCL patients^2–4^, about one third of patients with DLBCL succumbs to the disease eventually^5,6^. It is urgent to find new prognostic markers to identify those patients with very good or poor outcome and then give them more individualized therapy.

Based on the serum beta-2 microglobulin (Sβ_2_M) and serum albumin (SA) levels, the international staging system (ISS) has been used to evaluate the prognosis in multiple myeloma for many years^7^. However more and more evidence show that ISS is more related with the host features and immune system than multiple myeloma stage^8^. In this situation, ISS could also be used to predict outcome in the other hematological malignancies. Increasing studies show both Sβ_2_M and SA could predict outcome in DLBCLs^9–13^. However, to the best of our knowledge, the prognostic value of the combination of Sβ_2_M and SA in DLBCL has not been validated yet. Therefore, the purpose of the present study was to evaluate the prognostic significance of ISS in patients with DLBCL.

## Methods

In the present study, a total of 215 patients consecutively diagnosed as de novo DLBCL from January, 2001 to February, 2013 in Nanfang Hospital were reviewed. Patients with immunodeficiency-associated tumors and various types of DLBCL, including primary mediastinal, central nervous system, intravascular, testicular lymphomas, transformed NHL and posttransplant lymphoproliferative disorder were excluded from the study. All patients treated with cyclophosphamide, doxorubicin, vincristine, and prednisone (CHOP) or R-CHOP (rituximab plus CHOP) chemotherapy. The disease stage was evaluated according to the Ann Arbor staging system. Performance status was assigned according to the Eastern Cooperative Oncology Group (ECOG) scale. According to the ISS, all patients were divided into three groups: stage I group: β2-microglobulin <3.5 mg/L and serum albumin ≥35 g/L, stage II group: not stage I or stage III, stage III group: β2-microglobulin >5.5 mg/L. Electronic medical records of patients were collected prospectively and reviewed retrospectively in this study. All patients had provided written informed consent themselves or their guardians prior to treatment allowing the use of their medical records for medical research. This study was approved by the Ethics Committee of Southern Medical University affiliated Nanfang Hospital. All methods were performed in accordance with relevant guidelines and regulations.

Mann-Whitney test was applied to compare the differences between groups. Overall survival (OS) was calculated from the date of diagnosis to death from any cause or the last follow-up. Event-free survival (EFS) was calculated from the date of diagnosis to the date of documented disease progression, relapse or death from any cause. OS and EFS were estimated using the method of Kaplan-Meier and the log-rank test. Multivariable analysis was conducted by Cox proportional hazard regression model. All p values were two-sided and the significance was defined as p < 0.05. Statistical analysis was done using the Statistical Package of Social Sciences version 13.0 for Windows.

## Results

### Patients’ characteristics

A total of 215 de novo DLBCL patients were retrospectively analyzed. The male-to-female ratio was 1.90: 1 and the median age of patients at diagnosis was 49 years (range 19–80 years), which was similar to three other recent studies of Chinese DLBCL patients^14–16^, but much younger than those reported for DLBCL populations in the Western countries^17,18^. Based on the IPI score, 58 patients (27.0%) were in the intermediate-high or high-risk groups. Seventy-two patients (33.5%) had B symptoms and 61.4% patients had a SA ≥35 g/L. At baseline, 71.6% patients had a β2-microglobulin <3.5 mg/L, 15.3% had a level from 3.5 mg/L to 5.5 mg/L and 13.0% had a level >5.5 mg/L. According to ISS, 90 patients were in stage I group, 98 patients in stage II group and 27 patients in stage III group. Patients with high ISS risk tended to present with B symptoms (*p* = 0.011), high LDH (*p* = 0.019) and high IPI score (*p* = 0.001). Seventy-eight patients were treated with CHOP and other 137 patients were treated with R-CHOP. Baseline clinical features at the time of diagnosis are listed in Table 1.

**Table 1:** Patient characteristics according to ISS.

Characteristics	*N (%)*	ISS			*P-value*
		I (n = 90)	II(n = 98)	III(n = 27)	
Gender					0.688
Female	*141(65.6%)*	62*(68.9%)*	62*(63.3%)*	17*(63.0%)*	
Male	*74(34.4%)*	28*(31.1%)*	36*(36.7%)*	10*(37.0%)*	
Age(years)					0.609
<60 y	*175(81.4%)*	76*(84.4%)*	78*(79.6%)*	21*(77.8%)*	
≥60 y	*40(18.6%)*	14*(15.6%)*	20*(20.4%)*	6*(22.2%)*	
Systemic symptoms					0.011
A	*143(66.5%)*	70*(77.8%)*	56*(57.1%)*	17*(63.0%)*	
B	*72(33.5%)*	20*(22.2%)*	42*(42.9%)*	10*(37.0%)*	
IPI score					0.001
0–2	*157(73.0%)*	70*(77.8%)*	73*(74.5%)*	14*(51.9%)*	
3–5	*58(27.0%)*	20*(22.2%)*	25*(25.5%)*	13*(48.1%)*	
Performance status					0.239
0–1	*152(70.7%)*	66*(73.3%)*	71*(72.4%)*	15*(55.6%)*	
2–4	*63(29.3%)*	24*(26.7%)*	25*(27.6%)*	11*(44.4%)*	
Lactate dehydrogenase					0.019
Normal	*101(47.0%)*	*50(55.6%)*	*44(44.9%)*	*7(25.9%)*	
High	*114(53.0%)*	*40(44.4%)*	*54(55.1%)*	*20(74.1%)*	
Ann Arbor stage					0.082
I or II	*89(41.4%)*	44*(48.9%)*	38*(38.8%)*	7*(25.9%)*	
III or IV	*126(58.6%)*	46*(51.1%)*	60*(31.2%)*	20*(74.1%)*	
No. of extranodal sites					0.112
0–1	*113(52.6%)*	54*(60.0%)*	44*(44.9%)*	15*(55.6%)*	
≥2	*102(47.4%)*	36*(40.0%)*	54*(55.1%)*	12*(44.4%)*	
Cell-of-origin subtype					0.819
GCB	*48(22.3%)*	23*(33.3%)*	20*(33.9%)*	5*(26.3%)*	
NON-GCB	*99(46.0%)*	46*(66.7%)*	39*(66.1%)*	14*(73.7%)*	
Treatment					
CHOP	*78(36.3%)*	30*(33.3%)*	43*(43.9%)*	5*(18.5%)*	0.899
R-CHOP	*137(63.7%)*	60*(66.7%)*	55*(56.1%)*	22*(81.2%)*	

### ISS predicted survival in DLBCL patients treated with R-CHOP

The median follow-up was 52 months. The 5-years overall survival (OS) and event-free survival (EFS) were 80.5% and 63.9% respectively for all DLCBL patients. With respective to treatment, 78 patients were treated with CHOP and other 137 patients were treated with R-CHOP. The 5-years OS and EFS were 66.2% and 51.9% in patients treated with CHOP and 87.9% and75.8% in patients treated with R-CHOP.

**Figure 1:**
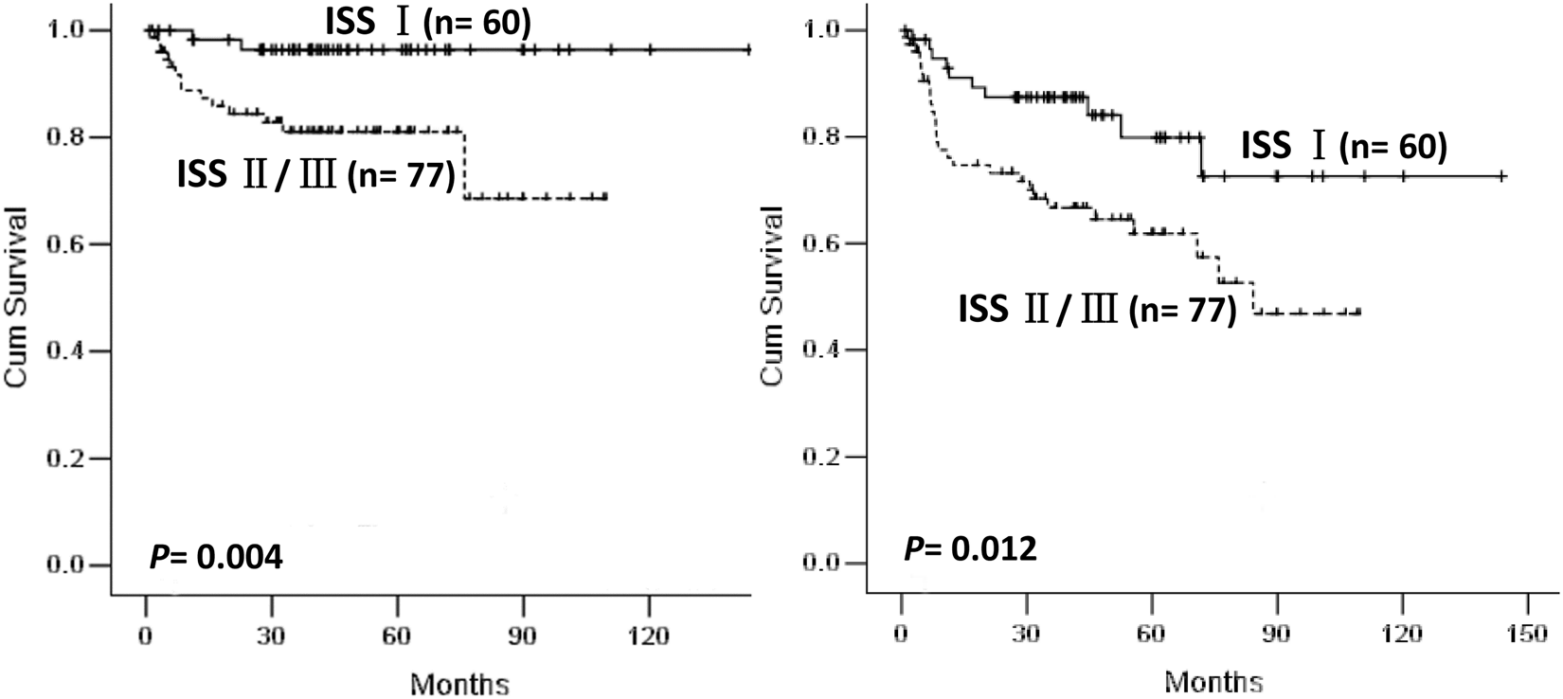
Kaplan-Meier curve for overall survival (**A**) and event-free survival (**B**) according to ISS for DLBCL patients treated with R-CHOP.

ISS showed no significant impact on OS and EFS in DLBCL patients treated with CHOP regimen (*p* = 0.906 and *p* = 0.972, respectively, Figure S1A–B). However, in the cases treated with R-CHOP, ISS could divide patients into different prognostic groups (*p* = 0.012, with 5-year OS of 96.4% versus 83.2% versus 75.8% for stage I, II and III respectively; *p* = 0.043, with 5-year EFS of 79.9% versus 60.8% versus 65.7% for stage I, II and III respectively, Figure S1C–D). Due to no significant differences were found between stage II and III groups, patients were re-divided into stage I and II/III groups with different prognosis (*p* = 0.012, with 5-year OS of 96.4% versus 81% for stage I, II/III respectively; *p* = 0.043, with 5-year EFS of 79.9% versus 61.8% for stage I, II/III respectively, Fig. 1) after incorporating stage II and stage III groups.

Multivariable analysis revealed that ISS, independent of IPI, indicated different survival in both OS (hazard ratio [HR] 5.690; 95% confidence interval [CI], 1.270–25.495, *p* = 0.023) and EFS (HR, 2.116; 95% CI, 1.005–4.455, *p* = 0.049) in DLBCL patients treated with R-CHOP. The multivariable survival analysis is shown in Table 2.

**Table 2:** Multivariable analysis of prognostic factors for survival.

Variable	OS			EFS		
	HR	95%CI	*P* value	HR	95%CI	*P* value
Covariates in the entire group (n = 215)						
IPI	1.340	0.983–1.826	0.064	1.234	0.974–1.563	0.082
ISS	1.822	0.921–3.604	0.085	1.419	0.873–2.307	0.158
Covariates in the R-CHOP group (n = 137)						
IPI	1.285	0.792–2.085	0.310	1.348	0.966–1.880	0.079
ISS	5.690	1.270–25.495	0.023	2.116	1.005–4.455	0.049

### ISS improved the IPI risk stratification in DLBCL patients

**Figure 2:**
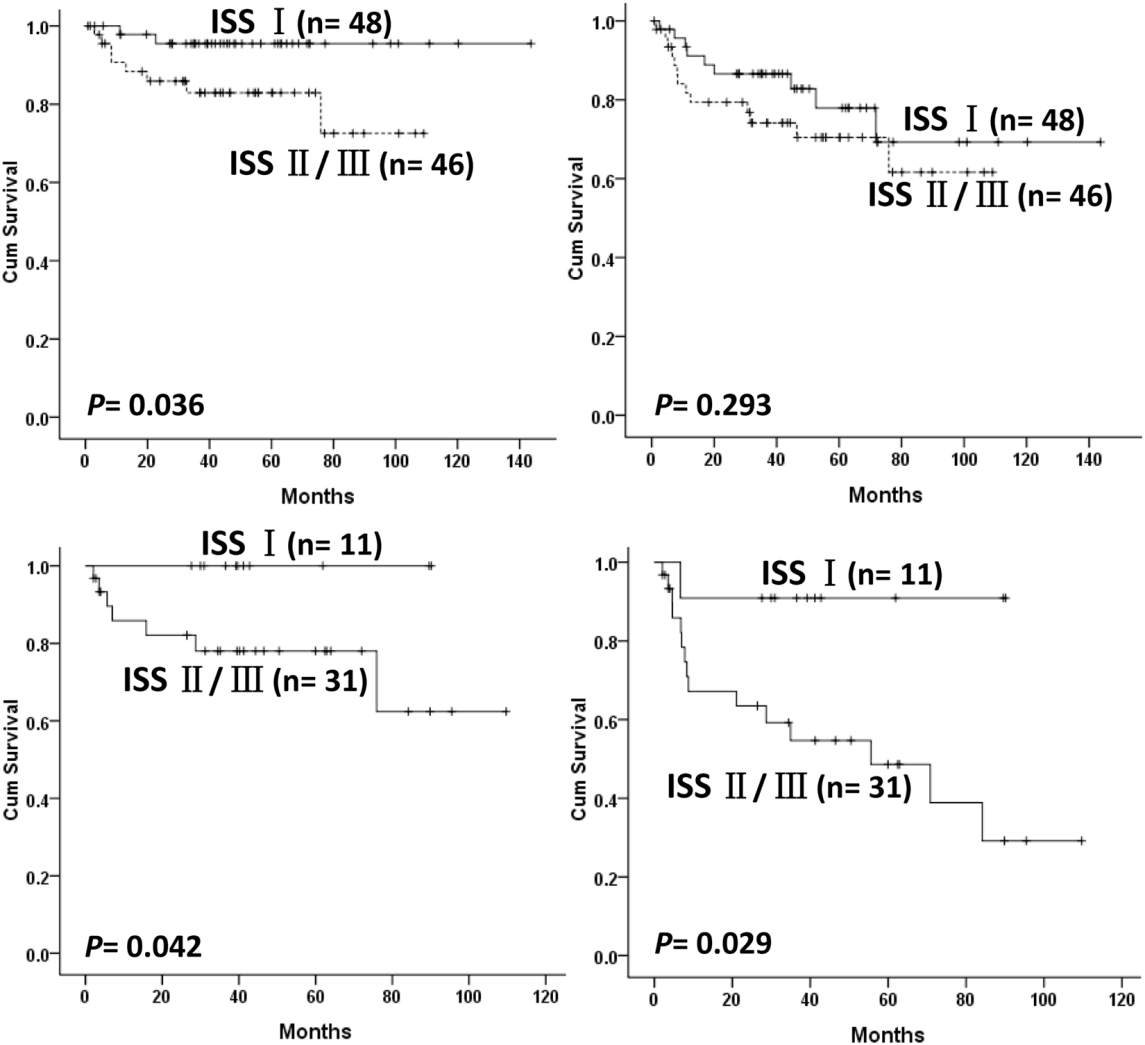
Kaplan-Meier curve for overall survival (OS) and event-free survival (EFS) in DLBCL patients treated with R-CHOP according to ISS. OS (**A**) and EFS (**B**) for low/ low intermediate risk IPI DLBCL patients (IPI = 0–2) according to ISS. OS (**C**) and EFS (**D**) for high intermediate/high IPI risk DLBCL patients (IPI = 3–5) according to ISS.

To further investigate whether ISS could improve the IPI risk stratification of DLBCL patients, we explored the prognostic value of ISS in low/low-intermediate and intermediate-high/high risk patients with DLBCL defined by IPI. ISS could identify a subgroup of patients with better outcome in intermediate-high/high risk patients (*p* = 0.042, with 5-year OS of 100% versus 78.0% for stage I and stage II/III, respectively; *p* = 0.029, with 5-year EFS of 90.9% versus 48.6% for stage I and stage II/III, respectively, Fig. 2A–B). However in low/low-intermediate risk patients, ISS could only identify patients with better overall survival (*p* = 0.036, with 5-year OS of 95.5% versus 82.9% for stage I and stage II/III, respectively), but not event-free survival (*p* = 0.293, with 5-year OS of 78.0% versus 70.5% for stage I and stage II/III, respectively, Fig. 2C–D).

## Discussion

ISS, defined by two routine and inexpensive pieces: Sβ_2_M and SA levels, is the most important prognostic system for multiple myeloma in the past ten years^7^. Recently more and more studies show both Sβ_2_M and SA could be used to predict outcome in DLBCLs^9–13^. But none of those studies have evaluated the prognostic value of the combination of Sβ_2_M and SA in DLBCL. In the present study, we retrospectively evaluated the prognostic significance of ISS in newly diagnosed DLBCL patients. To the best of our knowledge, this is the first time to provide evidence that advanced ISS stage at diagnosis was strongly associated with high-risk clinical features and indicated dismal outcome in DLBCL patients receiving rituximab-contained immunochemotherapy.

The underlying mechanisms by which ISS predicts DLBCL outcome are unclear. Decreased SA may be caused by the cytokine release by tumor cells such as IL-6 and the intense inflammatory response to the tumor, which may be a surrogate for a more aggressive behavior^19–21^. In this study, we also found that DLBCL patients with low serum albumin tended to present with aggressive clinical behavior including elevated LDH, B symptoms, advanced Ann Arbor stage and subsequent unfavorable outcome, which was consistent with previous studies^19^.

Increasing studies shows that elevated Sβ2 M is associated with dismal outcome in patients with DLBCL^12,13,22^. However the mechanisms for Sβ_2_M and outcome in DLBCL remain largely unknown. β_2_M is a subunit of the light chain of the class I major histocompatibility complex (MHC) and is released from the complex during cell turnover^23^. Elevated Sβ_2_M also implies the abnormal of the light and heavy chains of MHC class molecules, which can help cancer cells change epitope expression to escape T cell recognition^24,25^. Thus, Sβ_2_M may indicate cell turnover rate, high tumor burden and immune disorder in patients with DLBCL which is associated with survival.

Our study also showed IPI independent impact of ISS on the survival of DLBCL patients treated with rituximab-contained immunochemotherapy. This observation supports the involvement of ISS in immune system mediating the effect of rituximab and may provide further information on the mechanism of effect of rituximab. Further analysis showed ISS was able to identify a subset of very good outcome patients in high IPI risk DLBCL patients and if confirmed in further series could further improve risk stratification to guide treatment in DLBCLs. It is interesting that another study showed albumin can improve the low-risk NCCNIPI definition, in partially agreement with our findings. The addition of low serum albumin as a risk factor into NCCN-IPI augmented NCCN-IPI identifies more patients as low risk and maintains the excellent prognosis for this group compared to the conventional NCCN-IPI risk definition^26^.

It should be noted that this is a retrospective study with a relatively small number of patients from a single medical center. Therefore, the choice of patients might have been biased. Nevertheless, we only enrolled patients with de novo DLBCL treated with standard first-line chemotherapy in this study, and all electronic medical records for patients were collected prospectively, which can reduce bias to the least. In addition, we suggest prospective study of larger cohorts in multi-centers to be performed in order to confirm our findings.

In conclusion, our retrospective study evaluated the prognostic value of ISS at diagnosis in an independent cohort of de novo DLBCL. Although need to be confirmed prospectively, our data suggests that advanced ISS stage is associated with an inferior outcome in DLBCL patients treated with R-CHOP. ISS could identify a subgroup of DLBCL patients with superior outcome from high IPI risk subgroup. If confirmed in prospective clinical trials, these findings will have immediate clinical value, which can help to avoid intensive therapy in the high IPI risk subgroup of DLBCL patients.

## Electronic supplementary material

Figure S1
