# Publisher Correction: The international staging system improves the IPI risk stratification in patients with diffuse large B-cell lymphoma treated with R-CHOP

**DOI:** 10.1038/s41598-019-43124-7

**Published:** 2019-05-03

**Authors:** Xiaolei Wei, Xiaoxiao Hao, Lizhi Zhou, Qi Wei, Yuankun Zhang, Weimin Huang, Jialin Song, Ru Feng, Yongqiang Wei

**Affiliations:** 1 Department of Hematology, Nanfang Hospital, Southern Medical University, Guangzhou, China; 2 Department of Biostatistics, School of Public Health, Southern Medical University, Guangzhou, China

Correction to: *Scientific Reports* 10.1038/s41598-017-13254-x, published online 19 October 2017

In the original version of this Article, Lizhi Zhou was incorrectly affiliated with ‘Department of Hematology, Nanfang Hospital, Southern Medical University, Guangzhou, China’. The correct affiliation is listed below.

Department of Biostatistics, School of Public Health, Southern Medical University, Guangzhou, China

In addition, Qi Wei was incorrectly affiliated with ‘Department of Biostatistics, School of Public Health, Southern Medical University, Guangzhou, China’. The correct affiliation is listed below.

Department of Hematology, Nanfang Hospital, Southern Medical University, Guangzhou, China

These errors have now been corrected in the PDF and HTML versions of the Article.

