# Supplementary material for: The international staging system improves the IPI risk stratification in patients with diffuse large B-cell lymphoma treated with R-CHOP
Source: Sci Rep. 2017 Oct 19;7:13592. doi: 10.1038/s41598-017-13254-x (PMC5648852; doi:10.1038/s41598-017-13254-x)
Supplement: Supplementary file 1 — Figure S1 [file 41598_2017_13254_MOESM1_ESM.pdf]

The international staging system improves the IPI risk stratification in patients with diffuse large B-cell lymphoma treated with R-CHOP

Xiaolei Wei[1], Yongqiang Wei[1], Xiaoxiao Hao[1], Lizhi Zhou[2] , Qi Wei[1], Yuankun Zhang[1], Weimin Huang[1], Jialin Song[1] and Ru Feng[1,3]

1. Department of Hematology, Nanfang Hospital, Southern Medical University, Guangzhou, China.

2. Department of Biostatistics, School of Public Health, Southern Medical University, Guangzhou, China

3. Correspondence: Ru Feng, Department of Hematology, Nanfang Hospital, Southern Medical University, No. 1838 North Guangzhou Avenue, Guangzhou 510515, China;

E-mail：ruth1626@hotmail.com

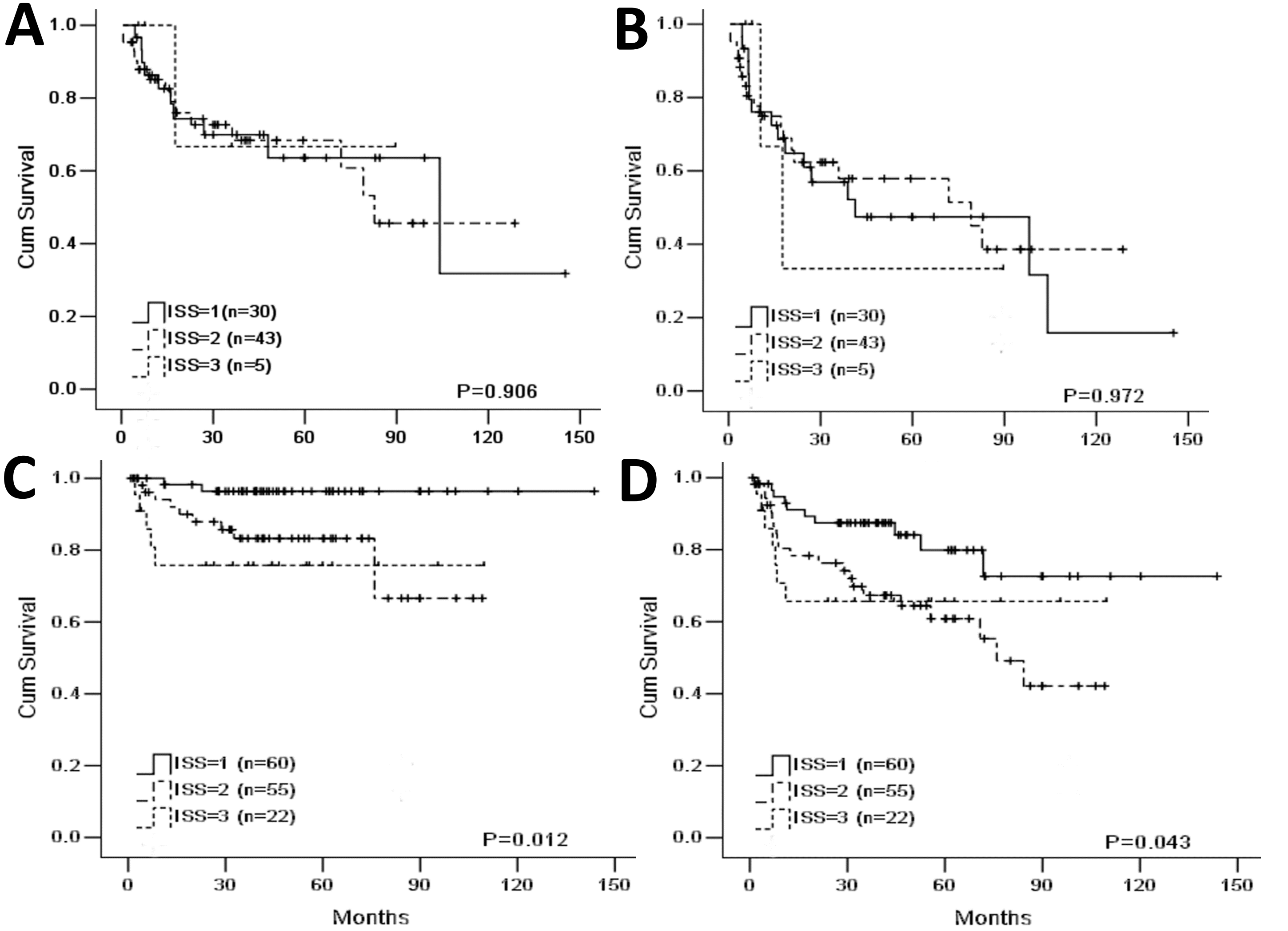

Figure S1. Kaplan-Meier curve for overall survival (OS) and event-free survival (EFS) in all DLBCL patients according to ISS. OS (A) and EFS (B) for DLBCL patients treated with CHOP according to ISS. OS (C) and EFS (D) for DLBCL patients treated with R-CHOP according to ISS.
